# Maternal SARS-CoV-2 infection in pregnancy disrupts gene expression in Hofbauer cells with limited impact on cytotrophoblasts

**DOI:** 10.1371/journal.ppat.1011990

**Published:** 2024-02-07

**Authors:** Elizabeth Ann L. Enninga, Huy Quang Quach, Jin Sung Jang, Maria Cristina Miranda de Araujo Correia, Yaroslav Fedyshyn, Bohdana Fedyshyn, Maureen Lemens, Dawn Littlefield, Supriya Behl, Elise Sintim-Aboagye, Maria C. Mejia Plazas, Maria C. Cardenas, Shree Chakraborty, Satoko Yamaoka, Hideki Ebihara, Akhilesh Pandey, Hu Li, Andrew D. Badley, Erica L. Johnson, Jie Sun, Andrew P. Norgan, Regan N. Theiler, Rana Chakraborty

**Affiliations:** 1 Department of Obstetrics and Gynecology, Mayo Clinic, Rochester, Minnesota, United States of America; 2 Department of Immunology, Mayo Clinic, Rochester, Minnesota, United States of America; 3 Mayo Clinic Vaccine Research Group, Department of Internal Medicine, Mayo Clinic, Rochester, Minnesota, United States of America; 4 Department of Laboratory Medicine and Pathology, Mayo Clinic, Rochester, Minnesota, United States of America; 5 Department of Molecular Pharmacology and Experimental Therapeutics, Mayo Clinic, Rochester, Minnesota, United States of America; 6 Children Research Center, Division of Pediatric Infectious Diseases, Department of Pediatric and Adolescent Medicine, Mayo Clinic, Rochester, Minnesota, United States of America; 7 Department of Molecular Medicine, Mayo Clinic, Rochester, Minnesota, United States of America; 8 Department of Laboratory Medicine and Pathology, Division of Clinical Biochemistry and Immunology, Mayo Clinic, Rochester, Minnesota, United States of America; 9 Center for Molecular Medicine, National Institute of Mental Health and Neurosciences, Bangalore, Karnataka, India; 10 Center for Individualized Medicine, Mayo Clinic, Rochester, Minnesota, United States of America; 11 Division of Infectious Diseases, Department of Medicine, Mayo Clinic, Rochester, Minnesota, United States of America; 12 Department of Microbiology, Biochemistry, and Immunology, Morehouse School of Medicine, Atlanta, Georgia, United States of America; 13 Thoracic Diseases Research Unit, Division of Pulmonary and Critical Care Medicine, Department of Medicine, Department of Immunology, Mayo Clinic College of Medicine and Science, Rochester, Minnesota, United States of America; 14 Carter Immunology Center University of Virginia School of Medicine, Charlottesville, Virginia, United States of America; University of Pittsburgh, UNITED STATES

## Abstract

**Background:**

Hofbauer cells (HBCs) and cytotrophoblasts (CTBs) are major cell populations in placenta. The indirect impact of maternal SARS-CoV-2 disease on these cells that are not directly infected has not been extensively studied. Herein, we profiled gene expression in HBCs and CTBs isolated from placentae of recovered pregnant subjects infected with SARS-CoV-2 during all trimesters of pregnancy, placentae from subjects with active infection, SARS-CoV-2 vaccinated subjects, and those who were unexposed to the virus.

**Methods:**

Placentae were collected within 4 h post-delivery and membrane-free tissues were enzymatically digested for the isolation of HBCs and CTBs. RNA extracted from HBCs and CTBs were sequenced using 150bp paired-end reads. Differentially expressed genes (DEGs) were identified by DESeq2 package in R and enriched in GO Biological Processes, KEGG Pathway, Reactome Gene Sets, Hallmark Gene Sets, and Canonical Pathways. Protein-protein interactions among the DEGs were modelled using STRING and BioGrid.

**Results:**

Pregnant subjects (n = 30) were recruited and categorized into six groups: infected with SARS-CoV-2 in i) the first (1T, n = 4), ii) second (2T, n = 5), iii) third (3T, n = 5) trimester, iv) tested positive at delivery (Delivery, n = 5), v) never infected (Control, n = 6), and vi) fully mRNA-vaccinated by delivery (Vaccinated, n = 5). Compared to the Control group, gene expression analysis showed that HBCs from infected subjects had significantly altered gene expression profiles, with the 2T group having the highest number of DEGs (1,696), followed by 3T and 1T groups (1,656 and 958 DEGs, respectively). These DEGs were enriched for pathways involved in immune regulation for host defense, including production of cytokines, chemokines, antimicrobial proteins, ribosomal assembly, neutrophil degranulation inflammation, morphogenesis, and cell migration/adhesion. Protein-protein interaction analysis mapped these DEGs with oxidative phosphorylation, translation, extracellular matrix organization, and type I interferon signaling. Only 95, 23, and 8 DEGs were identified in CTBs of 1T, 2T, and 3T groups, respectively. Similarly, 11 and 3 DEGs were identified in CTBs and HBCs of vaccinated subjects, respectively. Reassuringly, mRNA vaccination did not induce an inflammatory response in placental cells.

**Conclusions:**

Our studies demonstrate a significant impact of indirect SARS-CoV-2 infection on gene expression of inner mesenchymal HBCs, with limited effect on lining CTB cells isolated from pregnant subjects infected and recovered from SARS-CoV-2. The pathways associated with these DEGs identify potential targets for therapeutic intervention.

## Author summary

During the coronavirus disease 2019 (COVID-19) pandemic, our understanding of human immunity to SARS-CoV-2 infection and vaccination has increased markedly, but little is known about immunity against SARS-CoV-2 in pregnant subjects. In this study, we isolated Hofbauer cells and cytotrophoblasts from placentae of pregnant subjects infected with SARS-CoV-2 during all trimesters of pregnancy and profiled gene expression in these cells. Our gene expression analysis showed a significant and surprising impact of indirect SARS-CoV-2 infection on gene expression of inner mesenchymal Hofbauer cells, with limited effect on lining cytotrophoblasts isolated from pregnant subjects infected and recovered from SARS-CoV-2. Our data also support immunizing pregnant subjects with mRNA vaccination.

## 1. Introduction

Our knowledge of human immunity to COVID-19 has extensively improved since the first report of SARS-CoV-2 infection in late 2019 [1]. However, our understanding of and evidence for potential viral vertical transmission during maternal infection in gestation remains unclear. Although SARS-CoV-2 and viral RNA have been detected in placental tissue, there is limited evidence of transplacental transmission [2–6], with even fewer reports of exposed newborns acquiring infection in early postnatal life [7]. Only case reports published early in the pandemic documented vertical transmission of SARS-CoV-2 infection from pregnant subjects to their neonate [8–13]. Collectively, these observations suggest the placenta and humoral immunity in cord blood [14] are high effective but sometimes incomplete barriers against SARS-CoV-2 transmission in exposed newborns.

Even with the absence of vertical transmission, placental infection has been documented by a number of investigators [15,16] and meta-analyses have shown that COVID-19 during pregnancy is associated with preeclampsia, preterm birth, and stillbirth [17,18]. These observations are clinically supported by a large study from the Centers for Disease Control and Prevention (CDC), which showed that among >450,000 symptomatic COVID-19-infected women of reproductive age with known pregnancy status, admission to an intensive care unit, invasive ventilation, extracorporeal membrane oxygenation, and death were more likely among those who were pregnant than nonpregnant [19]. Since pregnant subjects are at higher risk of viral infection and severe complications [20–23], the negative impact of SARS-CoV-2 on placental cell types and function requires further investigation.

Hofbauer (HBCs) and trophoblast cells represent two major cell populations in placental tissue. HBCs are considered a key fetal immune macrophage population in the stroma of healthy placentae with a pivotal role in host defense [24]. While HBCs may be targets for several viruses at the maternal-fetal interface (MFI) [25,26], an expansion of HBC populations has been observed in response to SARS-CoV-2 placental infection [27]. At the MFI, syncytiotrophoblasts (STBs) may be more likely infected by SARS-CoV-2 than cytotrophoblasts (CTBs) reflecting direct contact with maternal blood [28]. In human placental explants, SARS-CoV-2 proteins and/or RNA were detected in both CTBs and STBs, and the magnitude of infectious virus correlated with ACE2 expression [29]. SARS-CoV-2 RNA was also detected in CTBs [30] and compared to HBCs and STBs, the former were more vulnerable to SARS-CoV-2 infection *in vitro* [31], which may be due to upregulation of receptors exhibiting tropism for CTBs [32]. However, even in placentae testing negative for SARS-CoV-2, vascular and inflammatory changes have been noted, suggesting this virus can have indirect consequences on placental function [33,34]. Since these cell populations dominate in number at the MFI, their properties during maternal infection and following vaccination need characterization *ex vivo*. Such studies may help elucidate the impact of SARS-CoV-2 infection in pregnancy on medium- and long-term outcomes in exposed fetuses and infants.

In this study, we enrolled 30 pregnant subjects at different stages of infection/vaccination with SARS-CoV-2 and characterized gene expression in HBCs and CTBs. Intriguingly, our results demonstrated that the numbers of potentially dysregulated genes in mesenchymal-derived HBCs within the villous core stroma were ~30 times greater than that measured in CTBs, which line the villous tree. We also describe differential gene regulation of HBCs associated with timing of infection during gestation.

## 2. Results

### 2.1. Clinical and demographic characteristics of study participants

**Fig 1:**
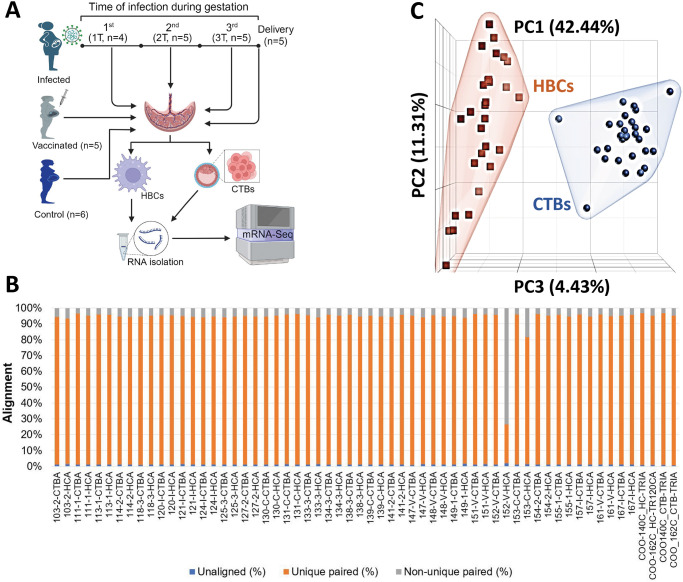
Study overview. A) Study design. Pregnant subjects (n = 30) recruited for this study were categorized into 6 groups based on the time of infection: i) Four participants were infected during the first trimester (Group 1T), ii) Five infected during the second trimester (Group 2T), iii) Five infected during the third trimester (Group 3T), iv) Five tested positive by PCR at delivery time (Group Delivery), v) Six never infected (Control group), and vi) Five fully mRNA-vaccinated by delivery (Vaccinated group). Placentae were collected within 4 h following delivery. Cytotrophoblasts (CTBs) and Hofbauer cells (HBCs) were isolated from placentae, subjected to RNA extraction and subsequent mRNA sequencing. Gene expression profiles in CTBs and HBCs were compared across six different groups. Illustrations of Fig 1A was created using BioRender. B) Breakdown alignment for all sequenced samples (n = 60). One Hofbauer cell (HBCs) sample, 152-V-HCA, did not pass QC and was removed from downstream analysis. Therefore, a total of 59 cell samples of HBCs (n = 29) and CTBs (n = 30) were included in the downstream analysis. C) Principal component analysis (PCA) plot after removing one HC sample that did not pass QC. The gene expression in HBCs and CTBs were visually separated.

Between June 2020 to August 2021, we conducted a cross-sectional cohort study of pregnant subjects of mostly European descent (77%), who were either infected with SARS-CoV-2 at different stages of gestation, vaccinated with the Pfizer mRNA vaccine, or not infected/not vaccinated during pregnancy. Pregnant subjects (n = 30) were recruited for this study who delivered at- or near-term, and were categorized into six groups: i) Four subjects were infected with SARS-CoV-2 in the first trimester (Group 1T), ii) Five were infected in the second trimester (Group 2T), iii) Five were infected in the third trimester (Group 3T), iv) Five tested positive by PCR at delivery (Delivery Group), v) Six were never infected (Control Group), and vi) Five were fully mRNA-vaccinated by delivery (Vaccinated Group). The experimental design of this study is schematically illustrated in Fig 1A. Clinical and demographic characteristics of the subjects are summarized in Table 1. The median age of the study cohort was 31 (range from 21 to 40 years). Participants in each group had a similar median gestational age of 39.1 weeks (Table 1). Infants of participants from each group had a median birthweight of 3.3 kg. Related clinical and demographic characteristics of participants across the 6 groups were comparable, which minimized confounding effects on gene expression profiles in isolated cell populations.

**Table 1:** Clinical and demographic characteristics of study participants. A total of 30 pregnant subjects were enrolled in this study and categorized into 6 different groups based on infection/vaccination status: i) Participants infected during the first trimester of gestation (Group 1T); ii) Participants infected during the second trimester (Group 2T); iii) Participants infected during the third trimester (Group 3T); iv) Participants testing positive for SARS-CoV-2 infection by PCR at the time of delivery (Delivery Group); v) Healthy participants without infection or vaccination (Control Group); vi) Participants fully mRNA-vaccinated by delivery (Vaccinated Group). In the “All” column, characteristics of all participants regardless of infection and vaccination status are summarized.

Characteristics	1T	2T	3T	Delivery	Control	Vaccinated	All
Group size	4	5	5	5	6	5	30
Median age (year, range)	32.5 (21, 36)	34 (24, 37)	28 (26, 35)	32 (21, 39)	29 (26, 31)	36 (29, 40)	31 (21, 40)
White (%)	50	60	80	80	100	80	77
Hispanic or Latino (%)	0	0	0	60	0	0	10
Median gestation age (week, range)	39.1 (39, 40)	39 (39, 39.6)	39.1 (34.1, 39.4)	39.1 (36, 41.4)	39.1 (39, 39.4)	39.1 (39, 39.7)	39.1 (34.1, 41.4)
Infant sex (% female)	75	20	20	20	33.3	60	36.7
Median infant weight (kg, range)	3.35 (2.6, 3.91)	3.49 (2.96, 3.59)	3.35 (3.12, 4.39)	3.57 (82.33, 3.92)	3.38 (2.83, 4.03)	3.03 (2.94, 4.15)	3.37 (2.33, 4.15)

mRNA-sequencing was performed on CTBs and HBCs isolated from term placentae. Following sample alignment, one HBC sample did not pass quality control and was therefore removed from subsequent analyses (Fig 1B). The remaining samples clustered strongly into HBCs (n = 29) and CTBs (n = 30) groups by principal component analysis (Fig 1C).

### 2.2. CTBs from subjects infected in the first trimester (Group 1T) had the highest numbers of up- and down-regulated genes compared to CTBs from the control group

**Fig 2:**
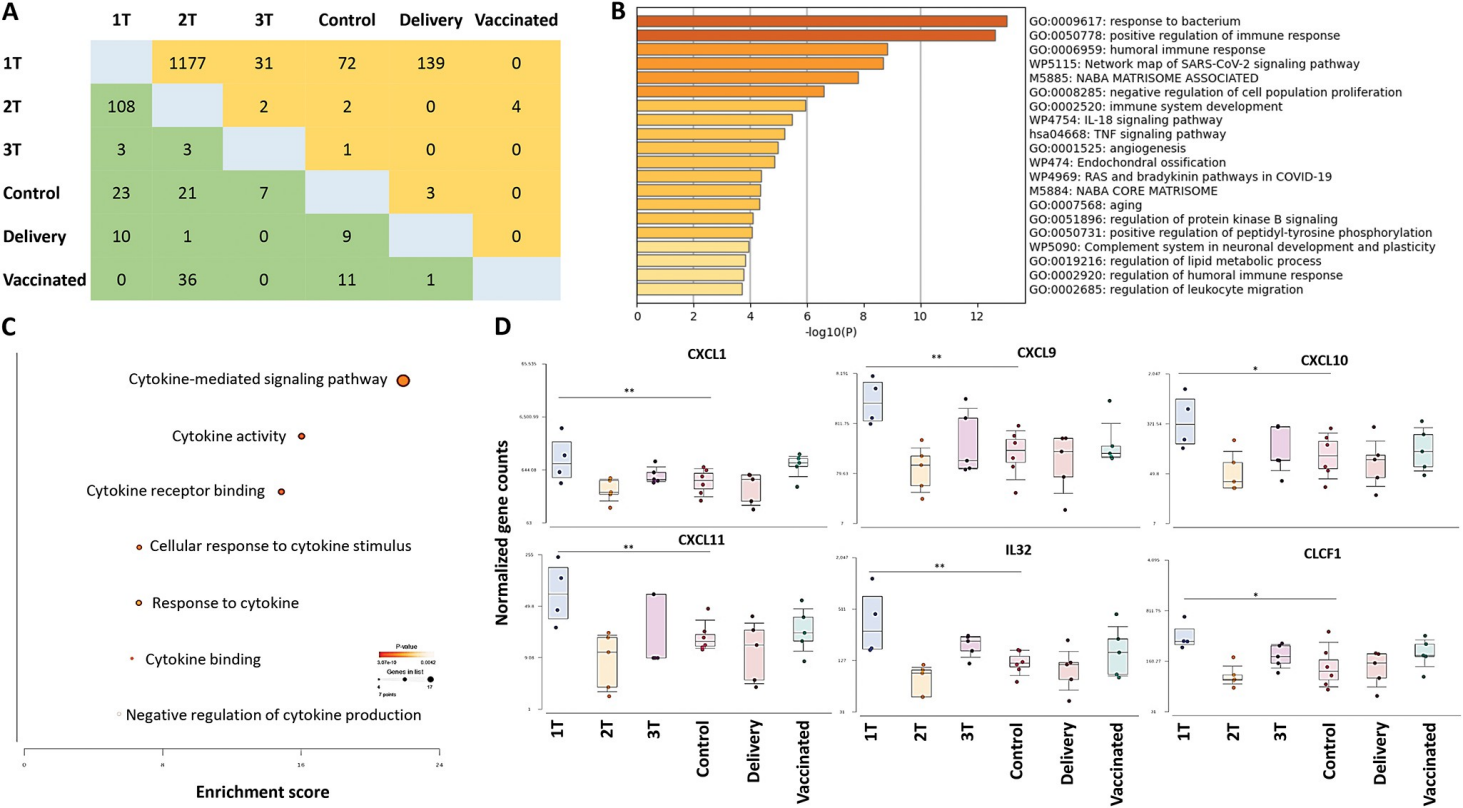
Gene expression profiles in CTBs. A) Number of differentially expressed genes (DEGs) in each comparison of CTBs across different groups. Orange = upregulated; Green = downregulated. (A) Enriched topology clusters, (B) and enriched biological pathways (C) of DEGs in Group 1T compared to the Control group. D) Normalized counts of six genes encoding CXCL1, CXCL9, CXCL10, CXCL11, IL-32, CLCF-1 across groups.

We observed that CTBs from subjects infected with SARS-CoV-2 in the first trimester (Group 1T) had the highest numbers of up- and down-regulated genes at delivery compared to CTBs from the Control Group (Fig 2A). Specifically, CTBs from Group 1T had 23 and 72 significantly down- and upregulated genes, respectively (Fig 2A). Of these potentially dysregulated genes, the top ontology clusters were related to immune response regulation, signaling, and development (Fig 2B and 2C). CTBs from Group 1T also significantly increased the expression of genes encoding chemokines and cytokines as compared to CTBs from the Control Group, including genes encoding chemokines critical for T cell (CXCL9/10/11) and neutrophil (CXCL1) trafficking as well as immune cell activation (IL-32 and CLCF-1) (Fig 2D).

### 2.3. Compared to CTBs, a significantly greater number of genes were altered in HBCs following maternal SARS-CoV-2 infection in pregnancy

**Fig 3:**
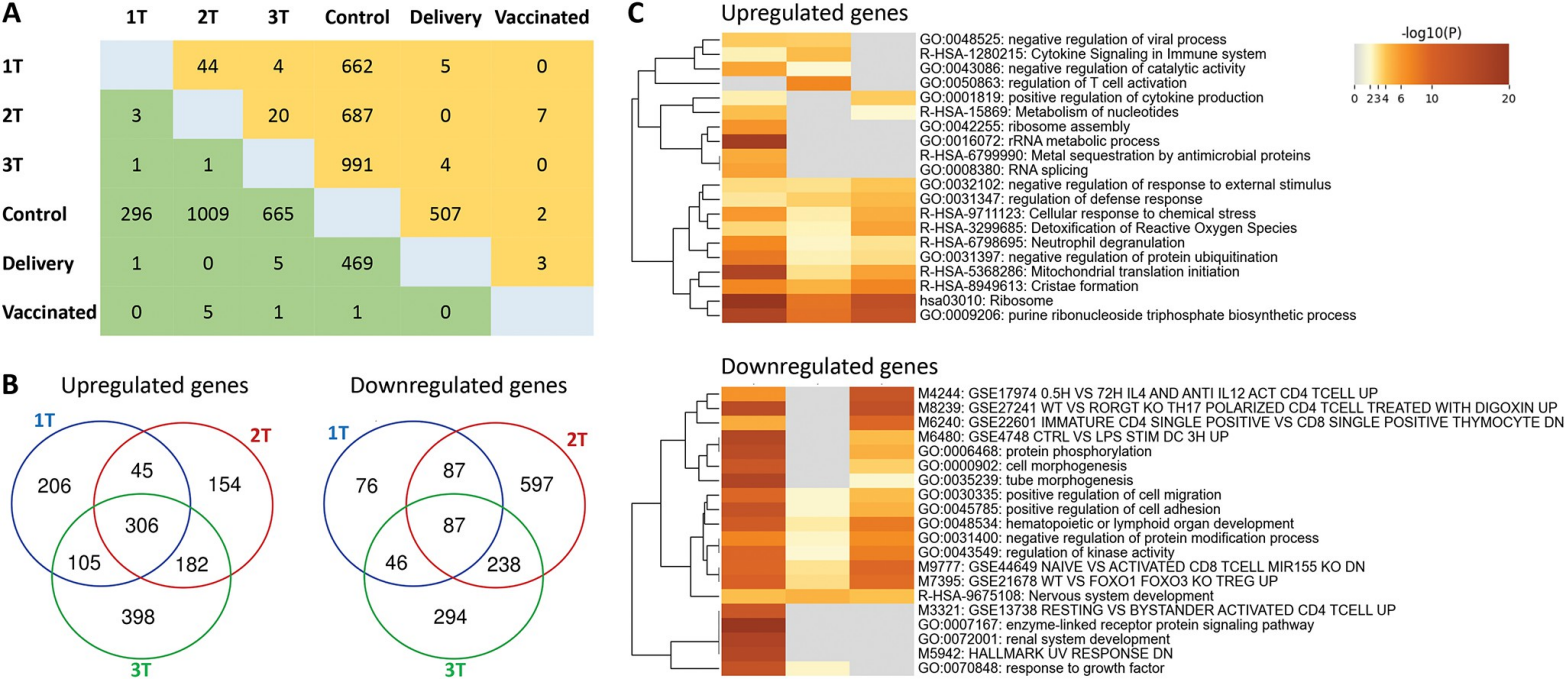
Gene expression profiles in term HBC infected across three trimesters (1T, 2T, 3T). A) Number of DEGs in each comparison of HBCs across different groups. Orange: upregulated; Green: downregulated. B) Overlapping DEGs (Up: upregulated genes; Down: downregulated genes) in HBCs from subjects infected in the first (1T), second (2T), and third (3T) trimesters. C) Enriched topology clusters of upregulated and downregulated genes.

We evaluated gene expression of HBCs in the different groups. Compared to CTBs, a significantly greater number of genes were altered in HBCs following maternal SARS-CoV-2 infection in pregnancy (Fig 3A). Similar to CTBs, vaccination led to very few changes in DEGs from HBCs (Fig 3A). Compared to the Control Group, HBCs from subjects infected at different time points during gestation (Groups 1T, 2T, and 3T) but recovered by delivery had significantly different numbers of DEGs, with infection in the second trimester (2T Group) having the highest number of DEGs (1696 in total with 687 up- and 1009 downregulated), followed by 3T Group with 1,656 DEGs (991 and 665 up- and downregulated genes, respectively) and T1 with 958 DEGs (662 and 296 up- and down-regulated genes, respectively) (Fig 3A). Of these DEGs, HBCs in Groups 1T, 2T, and 3T shared 306 up-regulated genes and 87 down-regulated genes (Fig 3B). Enriched ontology cluster analysis showed that these upregulated genes mapped to pathways involved in immune regulation for host defense, such as production of cytokines, chemokines, antimicrobial proteins, ribosomal assembly, and neutrophil degranulation (Fig 3C). Downregulated genes mapped with inflammation, morphogenesis, and cell migration/adhesion pathways (Fig 3C).

### 2.4. Gene expression in HBCs isolated from participants who tested positive for SARS-CoV-2 at delivery

**Fig 4:**
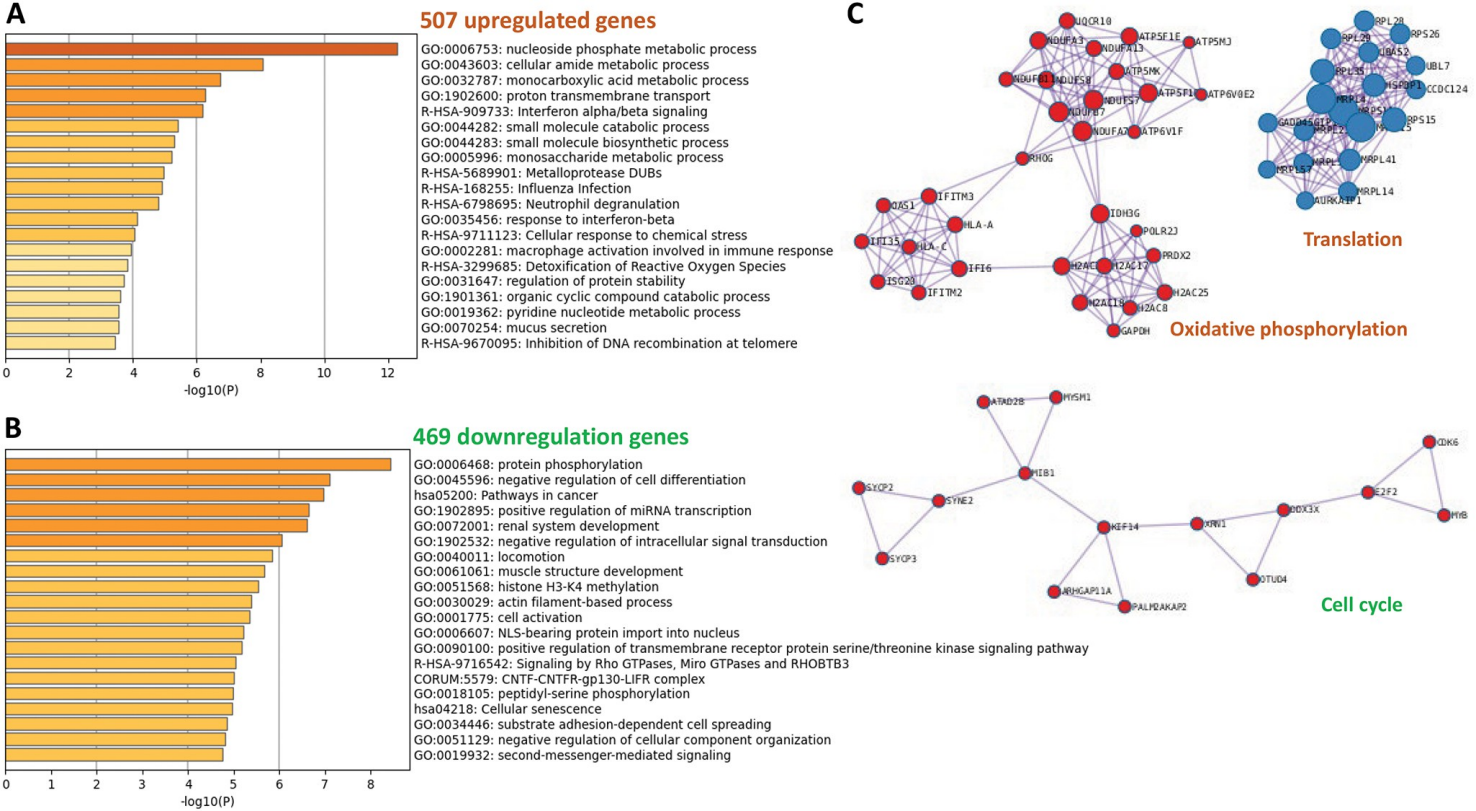
Gene expression profile of HBC isolated from subjects testing SARS-CoV-2 positive at delivery as compared to the control group. Enriched ontology clusters of (A) upregulated and (B) downregulated genes in HBCs. C) Protein-protein interaction network analysis demonstrated that these potentially dysregulated genes are involved in oxidative phosphorylation, translation, and cell cycle processes.

Compared to subjects from the Control Group, HBCs isolated from individuals who tested positive for SARS-CoV-2 at delivery (Delivery Group) had 507 and 469 up- and downregulated genes, respectively (Fig 3A). Enriched ontology cluster analysis showed that upregulated genes were involved in cellular metabolism, type 1 interferon responses and infection (Fig 4A). Downregulated HBC genes were associated with cellular differentiation, miRNA transcription, and signal transduction (Fig 4B). Protein-protein interaction network analysis demonstrated these genes participate in oxidative phosphorylation, translation, and cell cycle regulation (Fig 4C).

### 2.5. Gene expression Alterations in CTBs and HBCs during gestation

**Fig 5:**
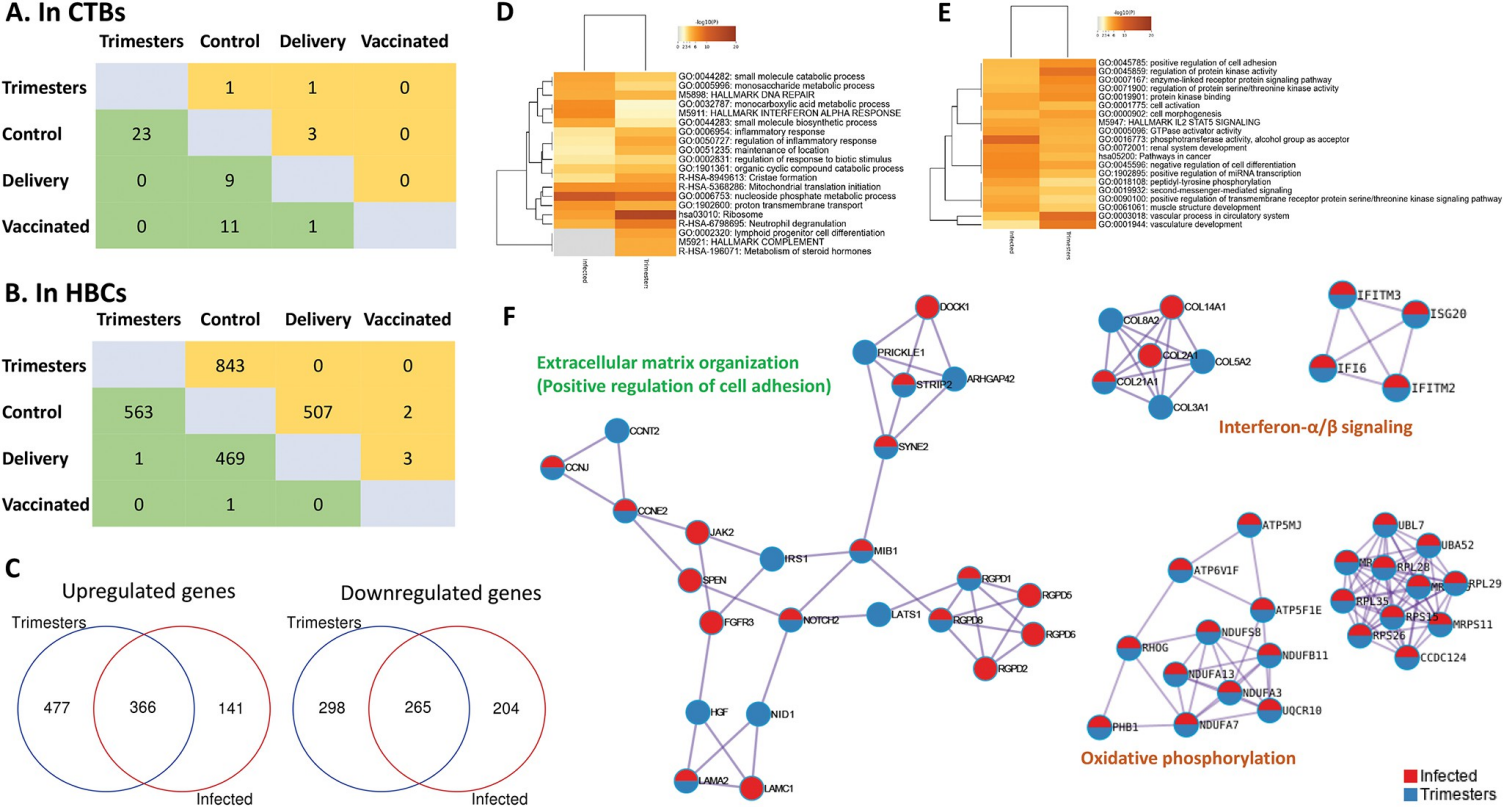
Changes in DEGs when grouping subjects infected in the first, second, and third trimester (Trimester group) compared to delivery, vaccinated and control groups. Number of DEGs in CTBs (A) and in HBCs (B). Orange: upregulated; Green: downregulated. C) Overlapping DEGs between HBCs isolated from the Trimester Group and Delivery group. Enriched ontology clusters of upregulated (D) and downregulated genes (E) in HBCs. F) Protein-protein interaction network of the significantly enriched biological pathways between Trimester versus Control groups and Infected versus Control groups.

We further grouped subjects infected during all three trimesters (Trimester group) who were recovered at delivery and compared their gene expression in both CTBs and HBCs with those in the Control, Delivery, and Vaccinated groups (Fig 5). Overall, there were very few genes altered in CTBs from these groups (Fig 5A), whereas many more genes were altered in HBCs (Fig 5B). Further analyses showed that HBCs in the Trimester and Delivery groups shared 366 and 265 up- and downregulated genes, respectively (Fig 5C). When comparing the clusters enriched in HBCs from the Trimester versus the Control Group, upregulated processes included innate immune responses, protein translation, and metabolism (Fig 5D). While there were some shared pathways, the upregulated and enriched processes in the Delivery Group demonstrated increases in metabolic and type I interferon responses. Downregulated processes which were enriched in the Delivery Group were related to cellular differentiation and phosphotransferase activity, while in HBCs from the Trimester Group, downregulated processes related to vascular development and cell adhesion (Fig 5E). Protein-protein interactions were also evaluated between HBCs exposed to SARS-CoV-2 at delivery versus HBCs isolated from subjects infected earlier in pregnancy and recovered prior to delivery. The top interactions were processes that regulate oxidative phosphorylation, translation, extracellular matrix organization, and type I interferon (IFN) signaling (Fig 5F).

**Fig 6:**
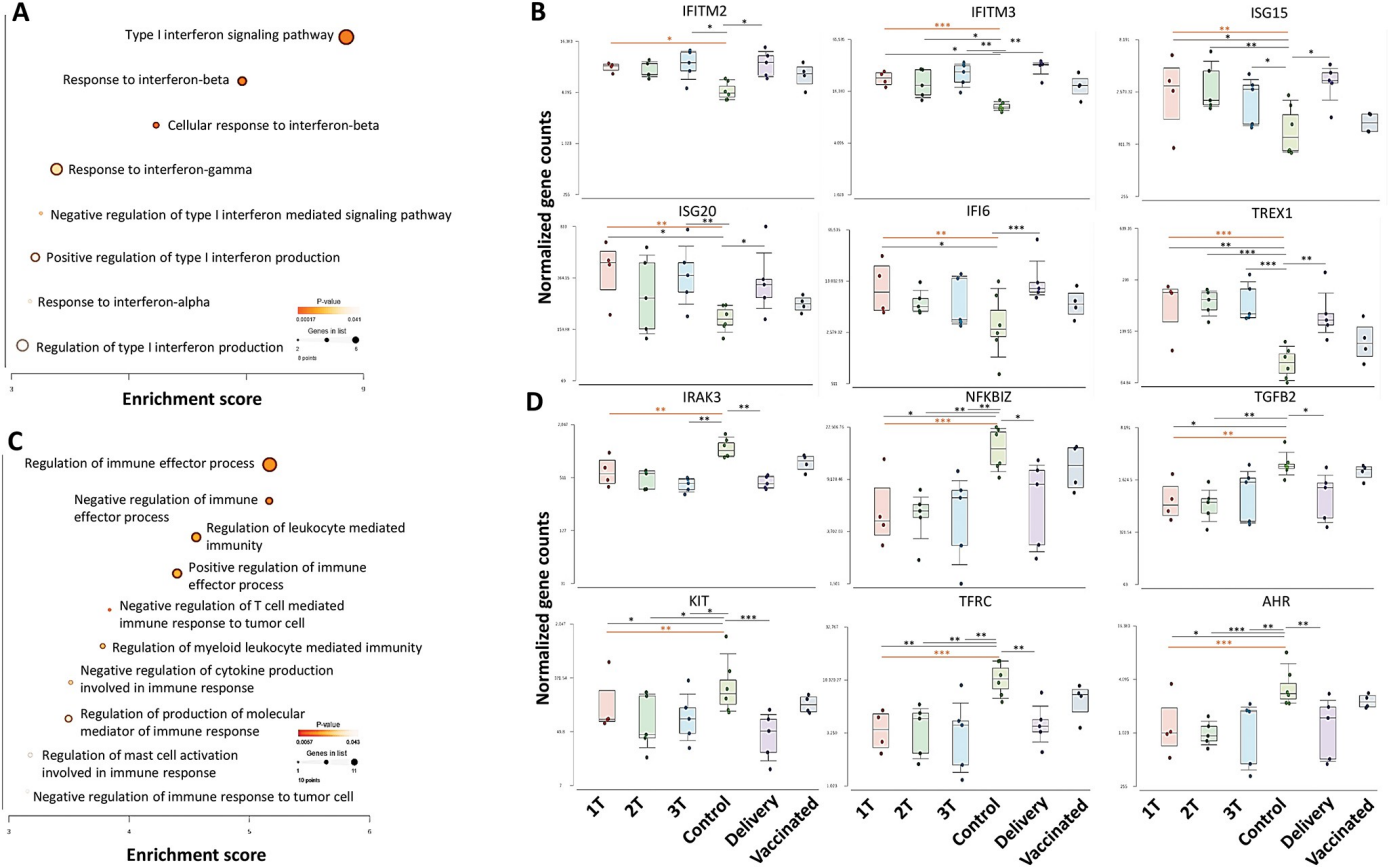
Gene enrichment of interferon signaling pathway between trimesters versus control group (A and B) and delivery versus control group (C and D) in HBCs. A) Enriched pathways of DEGs in HBCs of Trimester groups compared to Control group. B) The top 6 upregulated genes in Trimester groups compared to Control group. C) Enriched pathways of DEGs in HBCs of Trimester group compared to Control group. D) The top 6 downregulated genes in Delivery group compared to Control group.

Enrichment analysis confirmed significant changes in genes critical for type I IFN signaling as all enriched pathways were associated with interferon response, specifically cellular responses to IFN-β in HBCs of the Trimester Group (Fig 6A). The top 6 genes in the IFN pathway, including IFITM2, IFITM3, ISG15, ISG20, IFI6, and TREX1, were all upregulated compared to the Control group (Fig 6B). Meanwhile, DEGs from HBCs in the Delivery Group compared to Control Group, were mainly enriched in negative immune regulation pathways, such as negative regulation of immune effector processes and negative regulation of cytokine production involved in immune responses (Fig 6C). The top downregulated genes of these pathways, including IRAK3, NFKBIZ, TGFb3, KIT, TFRC, AHR are shown in Fig 6D.

## 3. Discussion

Despite extensive knowledge of SARS-CoV-2 immunity gained during the pandemic, the effects and consequences of COVID-19 on pregnant subjects and their exposed infants are poorly understood [35]. The MFI is composed of maternally-derived decidua and fetally-derived placenta containing chorionic villi with unique immune repertoires [36]. The maternal immune cells that populate the decidua include NK cells, macrophages, T cells, and dendritic cells [37], whereas the chorionic villous is composed of fetally-derived macrophages and invading maternal myeloid cells [38]. While abnormal placental pathologies, including inflammation and necrosis, are well documented [35,39], few studies have addressed how maternal SARS-CoV-2 impacts immunity in villous tissue within the fetal placental compartment [40–43]. In addition to placental inflammation, maternal SARS-CoV-2 infection is associated with altered cord blood immune cell composition including an increased frequency of memory T and B cells and nonclassical monocytes with maladaptive responses documented after immune stimulation [27]. Mild or asymptomatic maternal SARS-CoV-2 infection detected at delivery is also associated with an altered inflammatory milieu in the fetal circulation, including increased umbilical cord blood plasma levels of IL-1β, IL-6, IL-8, IL-18, IL-33, IFN-γ, caspase-1, nuclear factor of activated T cells, and CCL21 [44]. The functional and clinical implications of these phenotypic changes in the mother-infant dyad are unknown. Against this background, *ex vivo* studies have detected SARS-CoV-2 RNA in placental villi, including within maternal macrophages and HBCs [45], although vertical transmission has rarely been documented [8–13]. The inconclusive nature of these studies underscores the limited investigation of the impact of maternal SARS-CoV-2 infection on placental immune cells including in fetal villous tissue across gestation. In this study, we examined the impact of maternal COVID-19 in pregnancy by transcriptional analyses on paired cases and control samples. We compared and contrasted gene expression profiles in CTBs and HBCs isolated in placentae obtained from pregnant women infected at different time points in gestation (first, second, and third trimester, including those testing positive at delivery as a separate group). Uninfected and fully mRNA-vaccinated pregnant subjects were included as separate control groups.

Maternal SARS-CoV-2 infection in pregnancy was associated with marked increased basal activation of HBCs within chorionic villi in the fetal placental compartment. These data reflect observations from other investigators documenting that inflammation in the maternal systemic circulation may impact decidual tissue, which in turn could affect immune cells in placental fetal villous tissue [27,28]. Surprisingly, we found that mesenchymal-derived HBCs within the villous core stroma had ~30 times more DEGs than measured in CTBs, which line the villous tree. In a retrospective study of 22 placentae with documented chronic histiocytic intervillositis (CHI) and trophoblast necrosis, only 18.2% of the samples had identifiable SARS-CoV-2 in HBCs whereas 95.5% demonstrated viral positivity in trophoblast cells [40]. CHI is a rare but serious placental lesion which demonstrates CD68+ maternal macrophage infiltration into the villous space with subsequent perivillous fibrin deposition and trophoblast necrosis [46]. In the context of SARS-CoV-2 infection, this diagnosis is termed placentitis and is commonly associated with transplacental transmission and stillbirth [33,39,41,42]. Although we observed significant changes in gene expression of both CTB and HBC populations isolated from placentae during maternal COVID-19, we did not detect SARS-CoV-2 mRNA in raw sequencing data, suggesting an absence of the virus in these cells. Additionally, none of our cases were diagnosed with SARS-CoV-2 placentitis. In recent publications documenting the role of HBCs during maternal SARS-CoV-2 infection, expansion and activation of these cells was associated with elevated levels of myeloid cell recruiting chemokines [27,47,48]. Utilizing spatial transcriptomics, SARS-CoV-2 niches within the placenta have been described and shown to cause the depletion of M2 specific macrophages [43]. Our data may reflect HBC proliferation and hyperplasia, or specific HBC interactions with transplacental maternally-derived inflammatory proteins that have limited engagement with lining trophoblast cells. How and why HBCs within the villous core stroma are so dramatically affected compared to CTBs requires further investigation and could highlight novel mechanisms of viral associated CHI which could result in stillbirth.

Enriched ontology cluster analysis showed that upregulated genes from HBCs during maternal SARS-CoV-2 infection mapped to pathways involved in immune regulation for host defense including production of cytokines, chemokines, antimicrobial proteins, ribosomal assembly, and neutrophil degranulation. Moreover, gene expression profiles in HBCs isolated from participants testing positive for SARS-CoV-2 infection at delivery were involved in cellular metabolism, and infection. Protein-protein interaction network analysis demonstrated these genes participated in oxidative phosphorylation, translation, and cell cycle regulation. In addition, enrichment analysis confirmed significant changes in genes critical for type I IFN signaling and specifically cellular responses to IFN-β in HBCs. Key genes in the IFN pathway were all upregulated regardless of time of infection compared to the Control group. Collectively, our findings suggest that maternal SARS-CoV-2 infection in pregnancy stimulates innate immune responses by resident HBCs from chorionic villi. These data are not dissimilar to findings from Doratt and colleagues who documented increased expression of genes associated with migration, cytokine signaling, and apoptosis in HBCs obtained from placentae during mild/asymptomatic COVID-19 in unvaccinated pregnant subjects [27].

HBC are found in high abundance within the stroma of first trimester placental tissue [49]. Gene expression profiles in both CTBs and HBCs were dependent on the timing of infection in gestation. We identified more DEGs in HBCs infected at 1T than 2T or 3T, which may be associated with immune reprogramming in the fetal umbilical cord and infant peripheral blood in postnatal life. It has been well documented that innate cells, including macrophages, undergo metabolic and epigenetic changes after exposure to pro-inflammatory stimuli, which induces long-term reprogramming for future re-exposures [50]. This trained immunity has been shown in adults with SARS-CoV-2 infection where S-protein induces IL-1β release through the NLRP3 inflammasome resulting in long lived macrophage reprogramming and disease protection [51]. HBC responses to infection demonstrate changes in transcriptional profiles and cytokine release help to mediate pathogen elimination in the placenta to protect the fetus [52]; however, our understanding of these mechanisms requires much more investigation. In SARS-CoV-2-exposed fetuses and newborns, such alterations in HBCs may have short- or long-term impact on immunity. Whether these changes are associated with impaired or improved clinical responses to bacterial and viral infections in infancy and childhood remains to be determined. A limitation of our study was that none of the infants delivered to women with SARS-CoV-2 infection in pregnancy were longitudinally followed up. We could not therefore establish any correlation between gene expression in either CTBs or HBCs and clinical outcomes in SARS-CoV-2-exposed infants. Careful longitudinal clinical studies of SARS-CoV-2-exposed infants with appropriately matched controls will be essential to elucidate the immunologic and clinical impact of our molecular observations. Our findings of more DEGs in HBCs infected with SARS-CoV-2 at T1 may also reflect increased umbilical cord blood plasma levels of inflammatory cytokines and chemokines reported by other groups [44]. However, in our recently published data, although elevated levels of 28 cytokines/chemokines, mainly pro-inflammatory, were noted in maternal plasma with infection at delivery, umbilical cord blood plasma with maternal SARS-CoV-2 infection 2 weeks before delivery documented the emergence of anti-inflammatory cytokines [14].

There are several limitations in our study. First, there were co-morbidities, including asthma, obesity, hypertension, diabetes, cardiovascular, latent tuberculosis, in pregnant subjects across all six groups (S1 Table). These co-morbidities did not specifically occur in any groups, and we could not draw any conclusions on potential associations, if any, between these co-morbidities and gene expression. Second, in this study we focused on the differences in gene expression in HBCs and CTBs isolated from placentae of pregnant subjects infected at different trimesters as compared to control groups. We noted a difference in gene expression between pregnant subjects infected at 1^st^, 2^nd^, and 3^rd^ trimesters, but did not further examine this difference. Future studies should access the kinetics of gene expression in these cell populations isolated from pregnant subjects infected across three trimesters.

In summary, our study documents the significant indirect impact of SARS-CoV-2 infection on gene expression profiles in inner mesenchymal HBCs, with limited effect on lining CTB cells isolated from pregnant subjects during the three trimesters of pregnancy. These mainly inflammatory adaptations occurred in the absence of direct infection of these placental cells. We noted that maternal SARS-CoV-2 infection in pregnancy stimulates innate immune responses by resident HBCs from chorionic villi and that gene expression profiles in both CTBs and HBCs were dependent on the time of infection during gestation. The short and long-term impact of SARS-CoV-2 infection on immunity in the mother-infant dyad remains unclear but should prompt much needed longitudinal studies of exposed infants and children.

## 4. Materials and methods

### 4.1. Ethics statement

This cross-sectional, prospective study was approved by The Mayo Clinic Institutional Review Board (IRB# 20–003251). Written informed consent was obtained from each participant during enrollment.

### 4.2. Participant recruitment

Pregnant participants were recruited between June 2020 through August 2021 following a SARS-CoV-2 diagnosis, SARS-CoV-2 vaccination, or no infection/vaccination during gestation.

### 4.3. Isolation of Cytotrophoblasts (CTBs) and Hofbauer cells (HBCs)

Following delivery, the placenta was collected within 4 h for cell isolation and histologic evaluation. Membrane-free villous tissue (20 grams) was dissected for enzymatic cell isolation as previously described [53–56]. Briefly, tissues were thoroughly washed and mechanically dispersed in Hank’s balanced salt solution (HBSS) to wash out peripheral blood contamination. Minced tissue was subjected to three enzymatic digestions with Trypsin/DNase I at 37°C. For CTB isolation, the cell pellets from the Trypsin/DNase I digestion were re-suspended in DMEM/F12 with 10% FBS, 1mM L-glutamine, and 1% pen/strep and separated on a discontinuous gradient of Percoll [50%/45%/35%/30%] (GE Healthcare, Uppsala, Sweden) by centrifugation. Cells migrating to the 35%/45% Percoll interface were recovered and immunopurified by negative selection with simultaneous treatment using anti-CD9 and anti-CD45 antibodies to exclude platelets, smooth muscle cells, and immune cells (Miltenyi Biotech). The purity of CTBs was assessed by cytokeratin-7 staining (>97% as previously reported [55,56].

For HBC isolation, undigested tissue from the CTB isolation step was filtered through a 100 μm sieve and re-suspended in complete medium. This cell suspension was digested with collagenase for 1 h at 37°C. Digested tissue was then passed through a 70 μm and 40 μm cell strainer (BD Biosciences, Franklin Lakes, NJ). The mononuclear population was isolated by density gradient centrifugation on Histopaque-1077 (Sigma-Aldrich, St. Louis, MO), followed by CD14^+^ magnetic separation (Miltenyi Biotech, Germany).

### 4.4. mRNA sequencing (mRNA-Seq)

RNA was isolated from CTBs and HBCs using TRIzol reagents (Invitrogen). Nucleic acid concentration and purity were assessed using a Nanodrop (Thermo Fisher) and Bioanalyzer (Agilent). RNA integrity numbers (RIN) <7 were excluded from further analysis. The transcriptome was profiled by BGI (Shenzhen, China) using 150bp paired end reads with ≥30 million reads per sample.

### 4.5. Analysis of mRNA-Seq

FASTQ files were uploaded to Partek Flow software (Partek Inc., MO, USA) and primary QC was performed. The secondary analysis was performed using STAR (2.7.8a) aligner, which aligns reads to the human reference genome (hg38). The BAM files were quantified using the Partek E/M algorithm by Ensemble annotations (Ensemble Transcripts release 101) and then post aligned QC was performed [57]. A breakdown alignment for all samples is summarized in Fig 1B. One HBC sample from the Vaccinated group had a low unique paired alignment percent (26.6%), which was excluded from downstream analysis. The DESeq2 R package was used to identify differentially expressed genes (DEGs) between groups [58]. DEGs were identified using log2(fold change) ≥2 (for up-regulated genes) or ≤2 (for down-regulated genes) with a false discovery rate (FDR) <0.05. The Partek Flow software was used for generating PCA, volcano, and gene expression bar plots. Unsupervised hierarchical clustering was performed based on expression levels of the 631 shared genes between the Trimesters vs. Control groups and between the Delivery vs. Control groups, using the Euclidean distance and Average linkage approach.

### 4.6. Gene set enrichment analysis and functional annotation

Comparisons for each of the DEGs were made by Metascape (https://metascape.org) analysis for functional annotations and Partek Flow software for gene set enrichment analysis pipelines [59]. For generating the enrich ontology clusters, GO Biological Processes, KEGG Pathway, Reactome Gene Sets, Hallmark Gene Sets, Canonical Pathways, WikiPathways and PANTHER Pathway were used to identify statistically enriched terms, and then the top 20 most statistically significant terms within the cluster were chosen to represent the cluster (*p* <0.01, a minimum count of 3, and an enrichment factor >1.5). Partek Flow software was also used for gene set enrichment analysis using GO terms.

### 4.7. Protein-protein interaction enrichment analysis

The Metascape downstream analysis utilized processed enrichment analysis data to derive biological meaning from each MCODE network data [59]. The processed enrichment analysis data for each DEG enabled the identification of all protein-protein interactions (PPI), from which MCODE components were extracted and merged. The MCODE algorithm was then applied to identify densely connected network components for networks consisting of 3 to 500 proteins, enabling the identification of neighborhoods with high protein connectivity.

## Supporting information

S1 Table Comorbidities of pregnant subjects included in this study. (XLSX)
